# Quantification of 11β-hydroxysteroid dehydrogenase 1 kinetics and pharmacodynamic effects of inhibitors in brain using mass spectrometry imaging and stable-isotope tracers in mice

**DOI:** 10.1016/j.bcp.2017.12.013

**Published:** 2018-02

**Authors:** D.F. Cobice, D.E.W. Livingstone, A. McBride, C.L. MacKay, B.R. Walker, S.P. Webster, R. Andrew

**Affiliations:** a University/British Heart Foundation Centre for Cardiovascular Science, Queen’s Medical Research Institute, University of Edinburgh, 47 Little France Crescent, Edinburgh EH16 4TJ, UK; b Centre for Integrative Physiology, University of Edinburgh, Hugh Robson Building, 15 George Square, Edinburgh EH8 9XD, UK; c SIRCAMS, School of Chemistry, University of Edinburgh, Joseph Black Building, The King’s Buildings, West Mains Road, Edinburgh EH9 3JJ, UK; d Institute of Genetic Medicine, Newcastle University, International Centre for Life, Central Parkway, Newcastle upon Tyne NE1 3BZ, UK

**Keywords:** Corticosterone (PubChem CID: 5753), 11-Dehydrocorticosterone (PubChem CID: 5753), 11β-Hydroxysteroid dehydrogenase type 1, Glucocorticoid, Corticosterone, 11-Dehydrocorticosterone, Brain, Mass spectrometry imaging

## Abstract

11β-Hydroxysteroid dehydrogenase 1 (11β-HSD1; EC 1.1.1.146) generates active glucocorticoid hormones. Small molecule inhibitors have been developed to target 11β-HSD1 for the treatment of dementia; these must enter brain subregions, such as the hippocampus, to be effective. We previously reported mass spectrometry imaging measurement of murine tissue steroids, and deuterated steroid tracer infusion quantification of 11β-HSD1 turnover in humans. Here, these tools are combined to assess tissue pharmacokinetics and pharmacodynamics of an 11β-HSD1 inhibitor that accesses the brain.

[9,11,12,12-^2^H]_4_-Cortisol was infused (1.75 mg/day) by minipump for 2 days into C57Bl6 mice (male, age 12 weeks, n = 3/group) after which an 11β-HSD1 inhibitor (UE2316) was administered (25 mg/kg oral gavage) and animals culled immediately or 1, 2 and 4 h post-dosing. Mice with global genetic disruption of *Hsd11B1* were studied similarly. Turnover of d4-cortisol to d3-cortisone (by loss of the 11-deuterium) and regeneration of d3-cortisol (by 11β-HSD1-mediated reduction) were assessed in plasma, liver and brain using matrix assisted laser desorption ionization coupled to Fourier transform cyclotron resonance mass spectrometry.

The tracer d4-cortisol was detected in liver and brain following a two day infusion. Turnover to d3-cortisone and on to d3-cortisol was slower in brain than liver. In contrast, d3-cortisol was not detected in mice lacking 11β-HSD1. UE2316 impaired d3-cortisol generation measured in whole body (assessed in plasma; 53.1% suppression in rate of appearance in d3-cortisol), liver and brain. Differential inhibition in brain regions was observed; active glucocorticoids were suppressed to a greater in extent hippocampus or cortex than in amygdala.

These data confirm that the contribution of 11β-HSD1 to the tissue glucocorticoid pool, and the consequences of enzyme inhibition on active glucocorticoid concentrations, are substantial, including in the brain. They further demonstrate the value of mass spectrometry imaging in pharmacokinetic and pharmacodynamic studies.

## Graphical abstract

**Figure:**
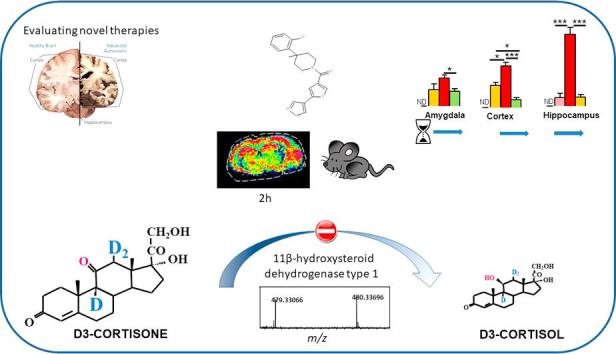

## 1 Introduction

Glucocorticoids act in many tissues and when present in excess can induce obesity, hyperglycaemia, and cognitive dysfunction. The reductase activity of 11β-hydroxysteroid dehydrogenase type 1 (11β-HSD1; EC 1.1.1.146) regenerates active glucocorticoid from inert keto-steroid substrates in glucocorticoid target tissues including liver, adipose tissue and brain. Whole body genetic disruption of 11β-HSD1 protects against the adverse systemic effects of high-fat diet, e.g. improving lipoprotein profile and glucose tolerance and attenuating weight gain, compared with wild-type mice [1]. Moreover, 11β-HSD1-deficient mice are protected against age-related cognitive decline, further substantiating the notion that reducing levels of glucocorticoids in tissues is of potential therapeutic benefit [2], [3].

Inhibitors of 11β-HSD1 have been developed to reduce tissue exposure to glucocorticoids in diseases such as Type 2 diabetes mellitus and Alzheimer’s disease [4]. However, the efficacy of 11β-HSD1 inhibitors in patients has been modest and inconsistent. In patients with type 2 diabetes mellitus, several 11β-HSD1 inhibitors displayed only moderate effects to improve glycaemic control [5], [6], [7], [8]. While debate still exists over whether 11β-HSD1 inhibitors have been tested in the correct populations [9], their clinical development as therapies for metabolic disease has largely been discontinued. In patients with Alzheimer’s disease, one study reported lack of efficacy of an 11β-HSD1 inhibitor but the data supporting pharmacodynamic engagement of the target in brain with this compound are contentious [10]. To justify progression of further candidate molecules for the treatment of dementia, such as UE2343 (Xanamem®) [11], it would be important to demonstrate that 11β-HSD1 contributes substantially to glucocorticoid regeneration in relevant tissues in vivo, and that 11β-HSD1 inhibitors have pharmacodynamic effects in these tissues and brain sub-regions.

Pharmacodynamic assessment of 11β-HSD1 is challenging since circulating steroid concentrations do not reflect the local tissue levels. Although active glucocorticoid levels may be decreased in tissue following 11β-HSD1 inhibition, circulating levels are normalized by feedback control of the hypothalamic-pituitary-adrenal axis; this is a recognised response to enhanced cortisol clearance following inhibition of cortisol regeneration in humans, evident in compensatory increases in ACTH levels and circulating adrenal androgens [5], [6], [7], [8], [11], [12]. Moreover, within the tissue pool of active endogenous steroid it is impossible to distinguish the proportion derived from the plasma pool from that regenerated intracellularly by 11β-HSD1. To address this in man we have developed an approach to trace steroid regeneration by 11β-reductase using stable-isotope labelled [9,11,12,12-^2^H]_4_-cortisol (d4F) [13]. d4F is converted to d3-cortisone (d3E) *in vivo* by loss of the 11-deuterium, providing a substrate for 11β-reductase to form d3F. The rate of formation of d3F reflects the reductase activity of 11β-HSD1, independently of adrenal synthesis. This approach has been used to quantify 11β-reductase activity in response to metabolic changes [14], [15], diet [16], [17], [18] and pharmaceutical agents [9] in man.

Access of drugs to tissues is commonly quantified by auto-radiography or by mass spectrometry. Auto-radiography has inherent non-specificity as the active drug cannot be distinguished from its radio-labelled metabolites. Measurement in tissue homogenates by mass spectrometry overcomes this problem, but lacks histological localisation. Mass spectrometry imaging (MSI) is increasingly being deployed as an alternative approach, albeit not for absolute quantitation [19]. It offers the advantages of simultaneously identifying both drug and metabolites in tissues while providing a fingerprint of pharmacodynamic changes in the metabolome of responsive organs [19]. Recently, we developed a novel approach to quantify steroid substrate and product ratios of 11β-HSD1 metabolites using MSI following steroid derivatization on tissue [20]. Here we report the combined application of stable-isotope tracer infusion with MSI to understand the pharmacodynamic responses to a pre-clinical tool molecule acting as a brain-penetrant 11β-HSD1 inhibitor, UE2316 [21], [22], [23], [24], [25], and demonstrate how these measurements can complement conventional measures of 11β-HSD1 activity *ex vivo*.

## 2 Materials and methods

### 2.1 Materials

[9,11,12,12-^2^H]_4_-Cortisol (d4F), [9,12,12-^2^H]_3_-cortisol (d3F) and internal standard [2,2,4,6,6,7,21,21-^2^H]_8_-corticosterone were from Cambridge Isotopes, MA, USA. Unlabeled steroids were from Steraloids Inc, PA, USA. Solvents were glass-distilled HPLC grade (Fisher Scientific, Loughborough, UK). UE2316, [4-(2-chlorophenyl)-4-fluoro-1-piperidinyl][5-(1*H*-pyrazol-4-yl)-3-thienyl]-methanone and UE2346 [26] were synthesized by High Force Ltd, Durham, UK [11]. Other chemicals were from Sigma-Aldrich (Dorset, UK) unless stated. Room temperature (RT) was 18–21 °C.

### 2.2 Animals and biomatrix collection

C57BL/6 mice (Harlan Olac Ltd, Bicester, UK) were studied under UK Home Office license. Groups of C57Bl/6 mice (n = 3/group, male, 12 weeks) were infused with d4F (1.75 mg/day, at a rate of 1.03 µL/h by surgically implanted sub-cutaneous mini-osmotic pumps, primed as per manufacturer instructions; ALZET model 1003, Cupertino, CA, USA) or vehicle (dimethylsulfoxide (DMSO): propylene glycol (PG) (50:50)). After 48 h mice were treated with UE2316 (11β-HSD1 inhibitor; 25 mg/kg oral gavage) or vehicle (2% DMSO, 38% PEG, 60% saline (0.9%), 5 mL/kg) and culled immediately or 1, 2 or 4 h post-dosing. Mice with genetic disruption of *Hsd11b1* (KO; male, 8–12 weeks [27] bred in-house on a C57Bl/6 genetic background) or C57Bl/6 controls were infused similarly with d4F or vehicle for 48 h.

Animals were killed by decapitation, plasma was prepared from trunk blood (collected in EDTA coated tubes) and tissues were snap-frozen in liquid nitrogen and stored at −80 °C.

### 2.3 MALDI-MS instrumentation and MS parameters

MSI was performed adapting the method described [20], using a 12T SolariX MALDI-FT-ICR-MS (Bruker Daltonics, MA, US) and employing a Smartbeam 1 kHz laser, operated with SolariX control v1.5.0 (build 42.8), Hystar 3.4 (build 8) and FlexImaging v3.0 (build 42).

The Girard T (GirT) derivative of d8-corticosterone (*m*/*z* 468.36718) was detected as before [20], but in CASI™ full mass (broadband) mode using an isolation window at 470 ± 25 Da in profiling mode, affording a 10–50-fold increase in sensitivity. The quasimolecular ion of UE2316 was monitored at +ve *m*/*z* 390.08377 and where appropriate, normalized to the matrix ion of α-cyano-4-hydroxycinnamic acid, +ve *m*/*z* 417.04834 Da. On-tissue spectral characterisation of the steroid tracers was performed by manual spotting (5 ng, 10 µg/mL; methanol: water (1:1) of d4F and d3F onto a control tissue section (murine brain). Ions were isolated at *m*/*z* 470.3 ± 20 Da for 30 s yielding a 2 Mword time-domain transient. The ions monitored for d4F-GirT and d3F-GirT were *m*/*z* 480.33696 and 479.33066 Da respectively. Structural confirmation of ions of GirT-d4F and GirT-d3F was assessed using LESA-nanoESI-FT-ICR-MS (Advion Tri-versa Nanomate) [20]. All analyses were carried out using 800 laser shots.

### 2.4 Quantitative analysis of UE2316

UE2316 was extracted from plasma (150 µL) enriched with internal standard, UE2346 (50 ng), into ethyl acetate (3 × 1.5 mL). The solutions were shaken (15 min) and, following centrifugation (3500*g*, 50 min, 4 °C), the organic extracts were dried under oxygen free nitrogen (OFN) at RT. Calibration curves were extracted from control plasma in the range 10–10,000 nM. UE2316 was extracted from liver and whole brain (∼150 mg, enriched with UE2346 (100 ng)) as previously described for brain ([20] Supplementary Information).

### 2.5 Metabolite identification

Metabolite identification was performed using a LESA-nanoESI coupled to FTICR-MS. Ions were isolated at 406.08 Da (±5) *m*/*z* (Metabolite III) and 404.06 Da (±5) (Metabolite IV) for 35 s yielding a 2 Mword time-domain transient and CE of 33 V was applied for CID experiments.

### 2.6 Analysis of corticosteroids in plasma and tissue extracts

#### 2.6.1 Plasma

Plasma analysis was performed as described previously [14]. Plasma d3E, d4F and d3F were quantified by LC/MS-MS. Epi-cortisol (10 ng) was added to 50 μL plasma and extracted using chloroform (0.5 mL). Solvent was evaporated and then reconstituted in mobile phase (95% water and 5% acetonitrile with 0.1% formic acid) before injection into an Acquity UHPLC (Waters, Manchester, UK) interfaced with a QTrap 5500 (Sciex, Warrington, UK) using a Sunfire C18 column (100 mm × 2.1 mm × 3.5 μm; Thames Restek, High Wycombe, UK, with column temperature 25 °C and mobile phase flow rate 0.5 mL/min). A gradient was employed between 10 and 20 min achieving 5% water and 95% acetonitrile. Ionization was achieved by positive electrospray. MS Source conditions were as follows: collision gas medium, ion spray voltage 5500 V, temperature 500 °C and curtain gas, GS1 and GS2 all 40 psi). The precursor and product *m*/*z* and MS parameters (declustering potential, collision energy, collision exit potential; V) were as follows: epi-cortisol (363 → 121; 131, 29, 14), d3F (366 → 121; 121, 27, 20), d4F (367 → 121; 121, 25, 20), corticosterone (347 → 121; 66, 69, 8), and d3E (364 → 77; 42 eV; 131, 29, 14). Compounds were quantified by the ratio of area under peak of interest to area under peak of internal standard against a standard curve (0.1–50 ng). In the absence of a commercial standard, d3E was calibrated against d4F.

#### 2.6.2 Liver

Murine liver (∼300 mg) was homogenised in methanol–acetic acid (100:1 v/v, 10 mL) using a mechanical homogeniser and assisted by ultrasonication and enriched with internal standard, d8-corticosterone (5 ng). The supernatant was retained and the pellet formed followed centrifugation (5000×*g*, 10 min, 4 °C), further extracted with methanol–acetic acid (100:1 v/v, 10 mL) and the supernatants combined and dried under oxygen free nitrogen (OFN) at RT. Residues were heated (40 °C, 60 min) with Gir T reagent (5 mg/mL in methanol with 0.2% trifluoroacetic acid; 10 µL) in an oven or water bath then allowed to cool at RT, mixed with α-cyano-4-hydroxycinnamic acid (CHCA; 10 µL; 10 mg/mL) in acetonitrile (80% + 0.2% v/v TFA) and analysed by MALDI-FTICR-MS as per 2.3, using an isolation window of 470.3 ± 50 Da.

#### 2.6.3 Whole brain

Steroids were extracted from whole brain as previously reported [20]. The residue was evaporated and derivatised as for liver (Section 2.6.2) and MALDI-FTICR-MS conducted as above (Section 2.3).

### 2.7 Tissue imaging of steroids in brain sections by MSI

#### 2.7.1 Tissue preparation for MS imaging

Brains were embedded in gelatin (50% *w/v)* and cryosections (10 µm) thaw mounted onto conductive indium tin-oxide (ITO)-coated glass slides (Bruker Daltonics, Bremen, GmbH), stored in a vacuum desiccator (RT, 1 h) and then at -80 °C. Adjacent sections were stained using haematoxylin and eosin. After fixation in cold acetone, tissue sections were examined using an optical microscope (40×, Leica Microsystems Inc, Bannockburn, IL, USA) with CCD camera (Hitachi, 3969, Japan). Internal standard, derivatization reagents and matrix were applied manually as described previously [20].

#### 2.7.2 Structural confirmation of GirT-d4F and GirT-d3F in a brain using LESA-nanoESI-FTIRMS

Brain sections were subject to derivatization with Gir T and then analysed immediately using LESA-nanoESI-FT-ICR-MS [20]. Ions were isolated at *m*/*z* 470.3 ± 20 Da for 30 s yielding a 2 Mword time-domain transient. CID fragmentation was carried out at 28 eV and CID spectra recorded.

### 2.8 Data analysis

For tissue homogenates, the average spectral intensities of the corresponding GirT-d4F, GirT-d3E, GirT-d3F and GirT-d8-corticosterone ions were presented as ratios of d4F/d3F, d4F/d8-corticosterone, d3F/d8-corticosterone and d3E/d8-corticosterone. The amount of internal standard (d8-corticosterone) was normalized (per mg tissue) across different tissues, to allow inter-tissue comparison. In MS images the average spectral intensities of GirT-d4F, GirT-d3E and GirT-d3F in regions of interest (ROIs) across the cortex, hippocampus and amygdala were presented similarly. An average value was calculated based on the weighted average. The intensities of GirT derivatives of deuterated steroids were corrected for any potential monoisotopic contribution of naturally occurring deuterium, assessed using standards. The rate of whole body appearance of d3F was calculated by dividing the infusion rate by the tracer/tracee ratio (d4F/d3F). Data are expressed as mean ± SEM and differences were analysed using a one-way or two-way ANOVA with Fisher’s LSD post hoc test and Mann-Whitney-Wilcoxon’s or Student’s *t*-test as appropriate. Statistical significance was accepted at p < 0.05. Signal to noise ratio is denoted as S/N.

## 3 Results

### 3.1 MS analysis of tracers

**Fig. 1:**
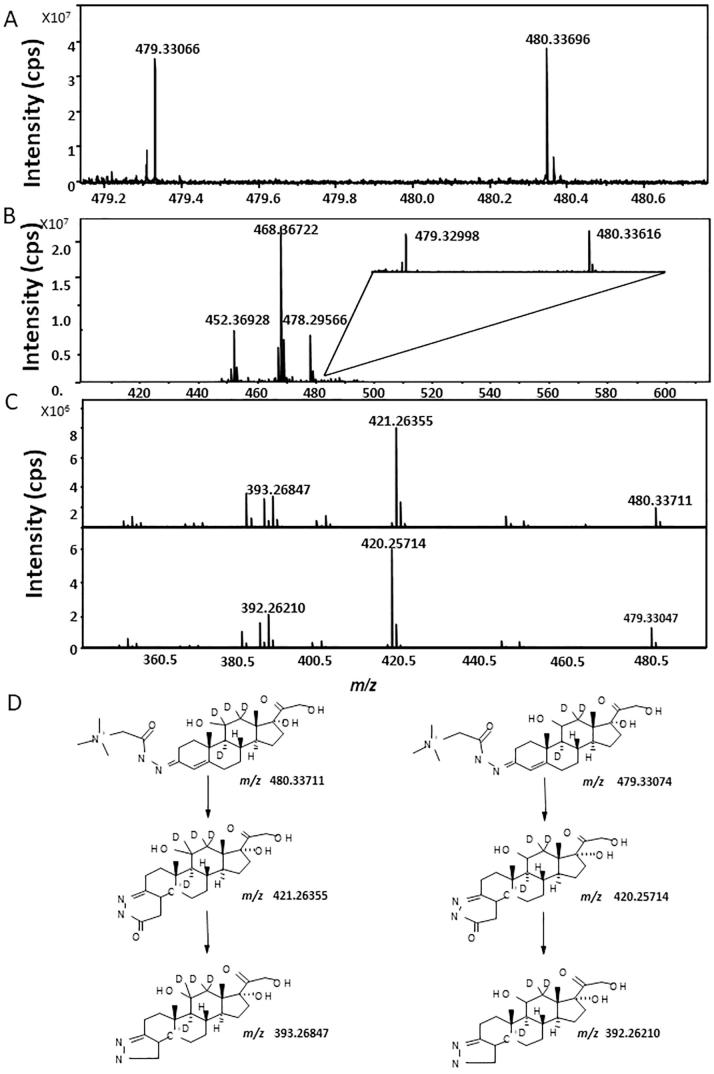
(A) Representative MALDI-FTICR-MS of Girard T (GirT)-derivatives of deuterium labelled glucocorticoid standards d4 and d3-cortisol (d4F, d3F, 5 ng). Observed ions at *m*/*z* 479.33066 Da for GirT-d3F and *m*/*z* 480.33696 Da for GirT-d4F. (B) Representative Mass spectrum of ions collated from murine brain from animals receiving d4-cortisol infusion following derivatization, showing ions with anticipated mass for Girard T derivatives of d4 and d3-cortisol. (C) Mass spectra of Girard T (GirT) derivatives of deuterium labelled cortisol tracers following liquid extraction surface analysis with nanoESI-FT-ICR collision induced dissociation (CID). CID mass spectra of precursors ions at *m*/*z* 480.33711 Da (GirT-d4F) and 479.33074 Da (GirT-d3F) and (D) proposed fragmentation patterns are shown. Cell isolation was 20 s and collision energy was set to 28 eV. cps: count per seconds.

d4F and d3F reacted with Gir-T and were detected at *m*/*z* 480.33696 Da and *m*/*z* 479.33066 Da respectively, with good mass accuracy (<10 ppm from their corresponding theoretical monoisotopic masses, *m*/*z* 480.33073 Da and *m*/*z* 479.33073 Da respectively; Fig. 1A). Tracer steroids were detected with similar mass accuracy when analysis of tissue from treated mice was performed (Fig. 1B). Structural confirmation by fragmentation was achieved using LESA-nanoESI-FT-ICR-MS, yielding fragmentation patterns typical of GirT hydrazones (Fig. 1C)[20], [28]. CID of the GirT derivatives (Fig. 1D) generated fragment ions characteristic of pyrazole derivatives formed by rearrangement of the derivatised group following loss of the quaternary amine tag [M−59]^+^ and carbon monoxide [M−87]^+^. These are characterised by ions at *m*/*z* 421.26355, *m*/*z* 393.26847 (GirT-d4F) and *m*/*z* 420.25714, *m*/*z* 392.25714 (GirT-d3F), respectively.

A commercial standard for d3E was not available. However, within tissues ions of *m*/*z* 477.32554 Da were detected within 5 ppm of the expected mass of the derivatised steroid in animals treated with d4F and not in controls.

### 3.2 Turnover of d4F in mice with genetic disruption of *Hsd11b1*

**Fig. 2:**
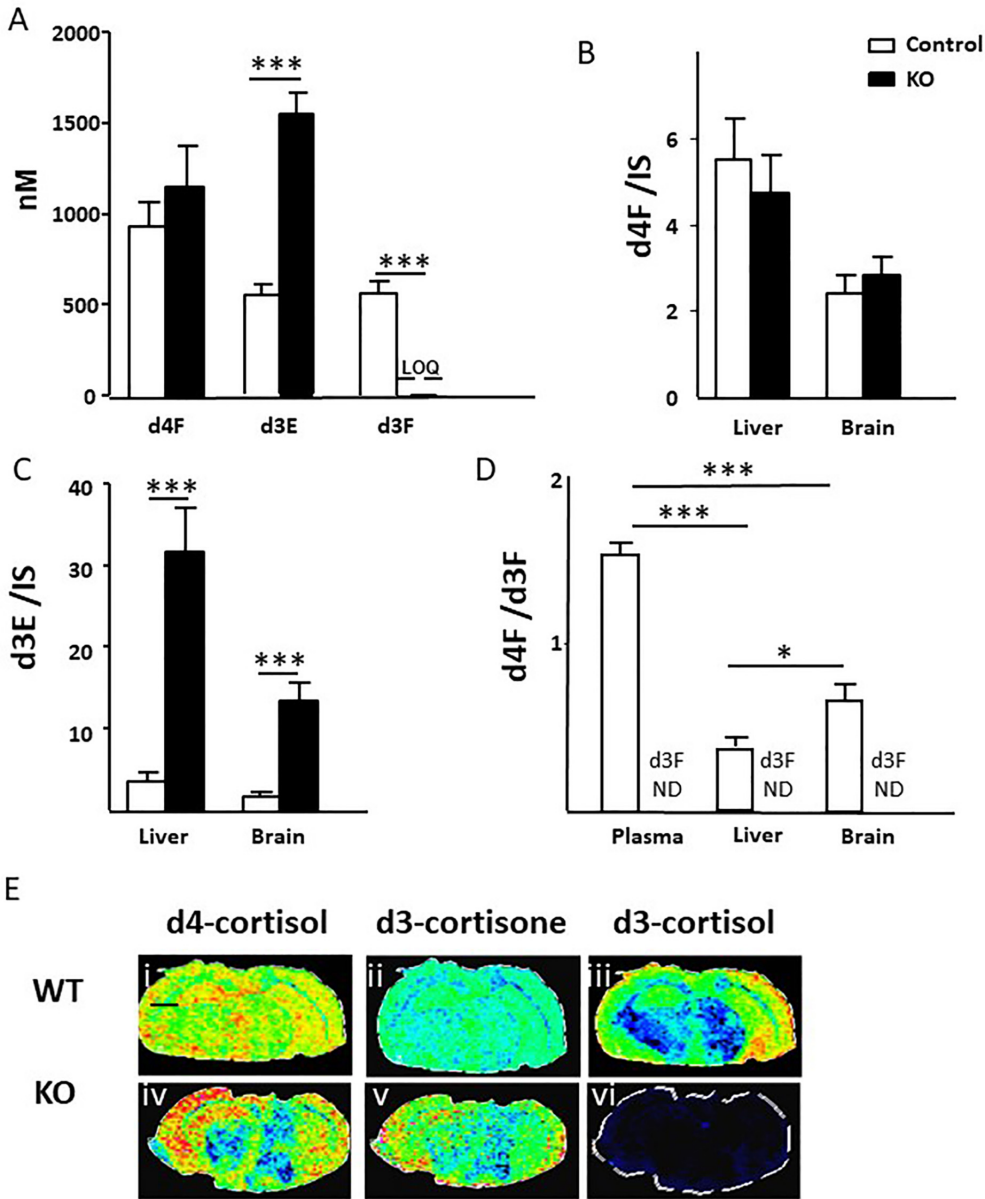
(A) Concentrations of d4-cortisol (d4F) were not different (p = 0.07) in mice with genetic disruption of 11β-hydroxysteroid dehydrogenase 1 (11β-HSD1) (KO) compared with their wild-type controls (WT). Concentrations of d3-cortisone (d3E) were higher in KO mice, whereas d3-cortisol (d3F) was not detected. Likewise (B) amounts of d4F in liver and brain expressed as a ratio to internal standard (IS) was not altered by disruption of 11β-HSD1, but (C) amounts of d3E were increased. (D) The d4F/d3F ratios were highest in plasma, lower in brain and even lower in liver (E) Mass spectrometry images of (i, iv) Girard T-d4F at *m*/*z* 480.33073 Da (ii, v) Girard T-d3E at *m*/*z* 477.29299 Da (iii, vi) Girard T-d3F at *m*/*z* 479.33073 Da in brains of WT and KO mice respectively, showing lack of regeneration of d3F following whole-body disruption of 11β-HSD1. Signal intensity is depicted by color on the scale shown. Scale bar (2 mm). Data are compared by Mann Whitney Wilcoxon, ^***^p < 0.001 and ^*^p < 0.05.

The circulating concentrations of d4F were the same between KO mice and controls, but d3E was approximately 3-fold higher in the plasma of KO mice (Fig. 2A). d3F was not detected in KO mice, but was present in controls, being generated with a rate of appearance of 121 ± 13 nmol/h. Within tissues, d4F was readily detected in liver and brain and amounts were not different between KO and controls (Fig. 2B). d3E was present in higher amounts in the livers and brains of KO mice compared to those from controls (Fig. 2C). The d4F/d3F ratio was lower in tissues than in plasma and lower in liver than in brain (Fig. 2D). d3F was not detected in any tissues in KO mice, as depicted in brain by MSI in Fig. 2E(vi).

### 3.3 Effects of a single dose of UE2316

#### 3.3.1 Pharmacokinetic analysis of UE2316

**Fig. 3:**
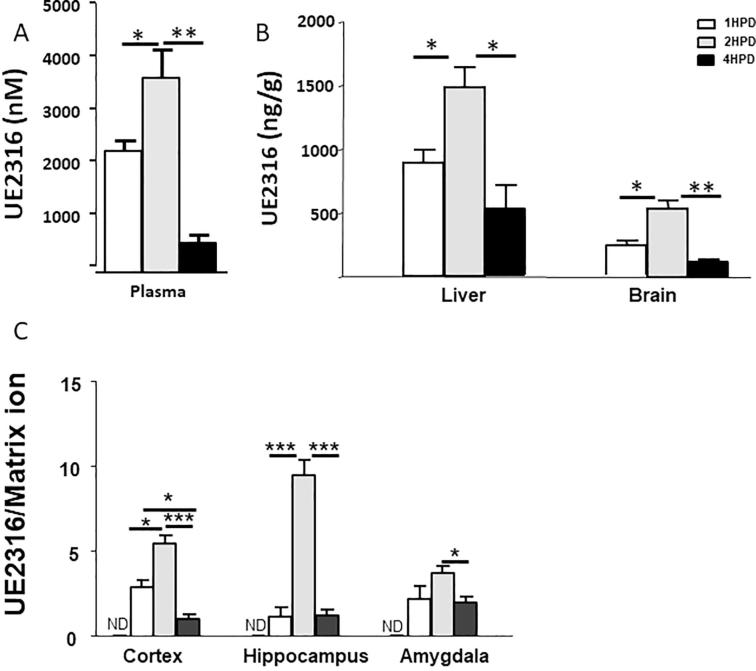
UE2316 levels in (A) plasma and (B) liver and brain in mice measured following a single oral dose. Drug levels reached a peak at 2 h post-dose (HPD) at all sites and were declining by the 4 h time point. (C) Amounts of UE2316 were quantified relative to the matrix cluster ion *m*/*z* 417.04834 Da by mass spectrometry imaging. The highest levels were found in hippocampus and all regions showed peak amounts at 2 h post-dose. Drug was not detected (ND) in brain regions of untreated animals. Statistical analysis was performed using a one-way ANOVA with Bonferroni's post hoc test. Data are mean ± SEM (n = 3). ^**^p < 0.01 and ^*^p < 0.05.

UE2316 was detected in plasma of mice culled at all time points 1, 2 and 4 h post-dose, peaking at 2 h (Fig. 3A). UE2316 penetrated liver and brain, reaching a peak at 2 h post-dose and significantly declining at 4 h, although still detectable. The amounts in liver exceeded those in brain, by approximately 2-fold at all time points. (Fig. 3B). Similar time-courses were observed in brain sub-regions (Fig. 3C), with highest levels of drug in hippocampus.

#### 3.3.2 MSI of UE2316 and its metabolites in tissues

**Fig. 4:**
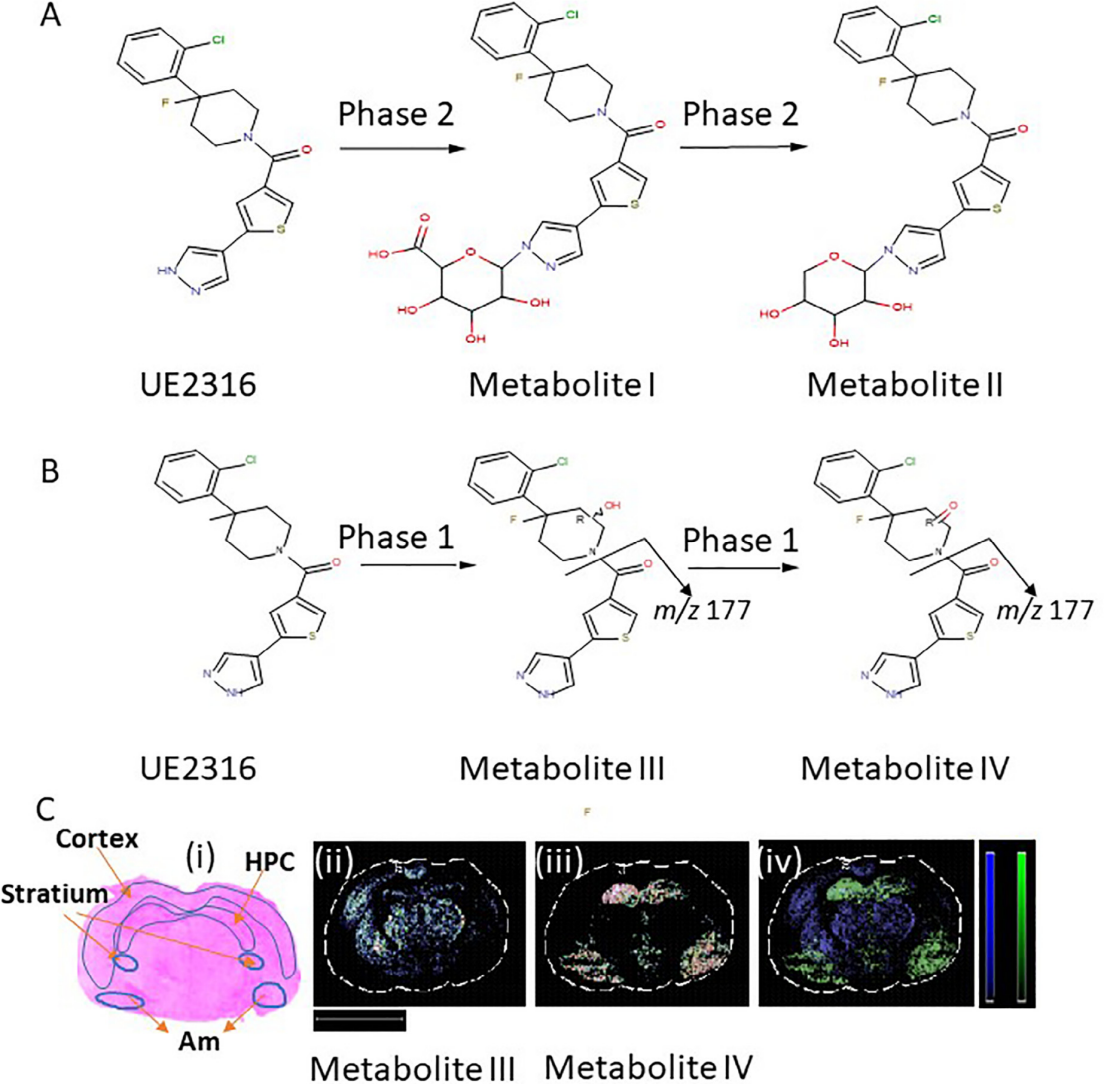
Proposed metabolic pathway of UE2316 in (A) liver and (B) brain. Metabolites were detected in murine brain by mass spectrometry imaging 4 h following single dose of UE2316. (C) Proposed Phase I metabolites had different distribution across brain, with metabolite III mainly distributed in the striatum (St) and cortex and metabolite IV found in the hippocampus (HPC) and amygdala (Am). (i) Haematoxylin and eosin histological staining. Molecular distribution maps of (ii) Potential hydroxylation metabolite (Metabolite III) at 406.07868 Da. (iii): Ketonic metabolite at *m*/*z* 404.06303 Da (Metabolite IV). (iv) Molecular superimposition maps of Metabolite III (blue) and Metabolite IV (green) show metabolites in different locations within the brain. Scale bar (2 mm). HPC: hippocampus, Am: amygdala, St: Striatum. (For interpretation of the references to color in this figure legend, the reader is referred to the web version of this article.)

Metabolites of UE2316 were identified in liver and brain from mice 4 h post-dose after treatment with UE2316 and structures proposed with mass accuracy calculation within 5 ppm of their corresponding theoretical monoisotopic masses. Two potential phase II metabolites were only observed in liver; Metabolite I was proposed at *m*/*z* C_25_H_27_ClFN_3_O_7_S 569.13211 Da potentially formed by N-glucuronidation and Metabolite II C_24_H_25_ClFN_3_O_5_S with *m*/*z* 521.01143 Da was suggested as a further phase II metabolite formed by further decarboxylation (Fig. 4A, with images in Fig. 7A).

In both brain and liver, two further potential metabolites were detected, with a fragment ion of *m*/*z* 177.01210 (C_8_H_5_N_2_OS; theoretical 177.01171). In the brain these had distinct spatial distributions. From the molecular structure of UE2316, it is anticipated that the piperidine alpha hydrogens will be prone to oxidation by Phase I CYP450 enzymes leading to potential hydroxylation and further oxidised ketone products (Fig. 4B) and fragmentation occurred at the amide bond. The proposed hydroxylation product at *m*/*z* 406.07868, Metabolite III (C_19_H_18_ClFN_3_O_2_S) (Fig. 4Cii) was detected mainly distributed across the striatum and cortex. The further oxidised metabolite at *m*/*z* 404.06303, Metabolite IV (C_19_H_16_ClFN_3_O_2_S, possible ketone formation) (Fig. 4Ciii) was mainly found in the hippocampus and amygdala. These metabolites were in distinct locations depicted by lack of superimposition (Fig. 4Civ).

#### 3.3.3 Pharmacodynamic assessment of 11βHSD1 activity

**Fig. 5:**
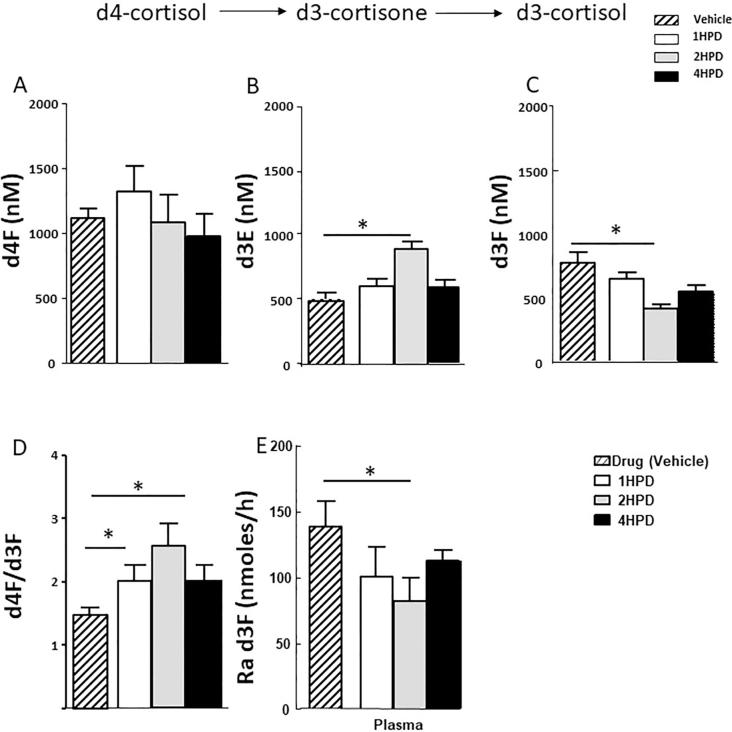
Concentrations of (A): d4-cortisol (d4F) (B) d3-cortisone (d3E) and (C) d3-cortisol (d3F) in plasma following a single dose of UE2316, an inhibitor of 11β-hydroxysteroid dehydrogenase 1. D4F did not change during the 4 h time period but concentrations of d3E increased 2 h post-dose and those of d3F reduced. (D) The d4F/d3F ratio increased again until 2 h reflected in a (E) reduced rate of appearance (Ra) of d3F, indicative of enzyme inhibition. Statistical analysis was performed using a one-way ANOVA with Bonferroni’s post hoc test. Data are mean ± SEM (n = 3). ^*^p < 0.05.

**Fig. 6:**
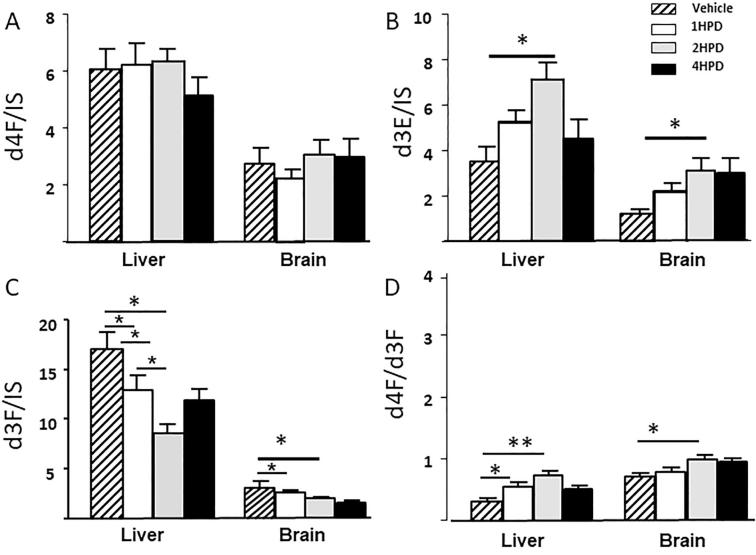
Amounts of (A) d4-cortisol (d4F) (B) d3-cortisone (d3E) and (C) d3-cortisol (d3F) in liver, and whole brain 4 h following a single dose of the 11β-hydroxysteroid dehydrogenase 1 inhibitor, UE2316. (D) Regeneration of d3F, assessed by d4F/d3F ratio, was detected in liver and brain and was suppressed by the inhibitor. Inhibition was sustained longer in brain than liver. Data are mean ± SEM (n = 3) and were compared by a one-way ANOVA with Bonferroni’s post hoc test. ^**^p < 0.01 and ^*^p < 0.05.

The extent of inhibition of 11β-HSD1 by UE2316 with time was assessed by tracer kinetics, measuring the enrichment of infused tracer with newly generated d3F (d4F/d3F) and rate of appearance (Ra) of d3F. Circulating d4F levels did not change (Fig. 5A) but accumulation of d3E was observed 2 h after UE2316 administration (Fig. 5B), with a concomitant reduction in circulating d3F (Fig. 5C), increased d4F/d3F ratio (Fig. 5D), and a corresponding 53.1% reduction in whole-body Ra d3F (Fig. 5E). These effects waned by 4 h. d4F tracer was detected in liver and whole brain after 48 h infusion (Fig. 6A); amounts were higher in liver than in brain. The increase in d4F/d3F ratio (indicative of enzyme inhibition) across the 4 h time course was greater in liver and brain than in plasma (p = 0.01); at the 2 h time point the ratio was increased approximately 2.3-fold in liver and 1.8-fold in brain and 1.5-fold in plasma (Fig. 6D). In liver, the levels of d3F declined (Fig. 6C) to a nadir 2 h post-UE2316, with accumulation of d3E (Fig. 6B). This corresponded with a reduced rate of formation of d3F, inferred from an increased d4F/d3F ratio (Fig. 6D). This effect had started to diminish by 4 h. In brain, a similar effect was observed 2 h post-UE2316 but inhibition was sustained until 4 h.

**Fig. 7:**
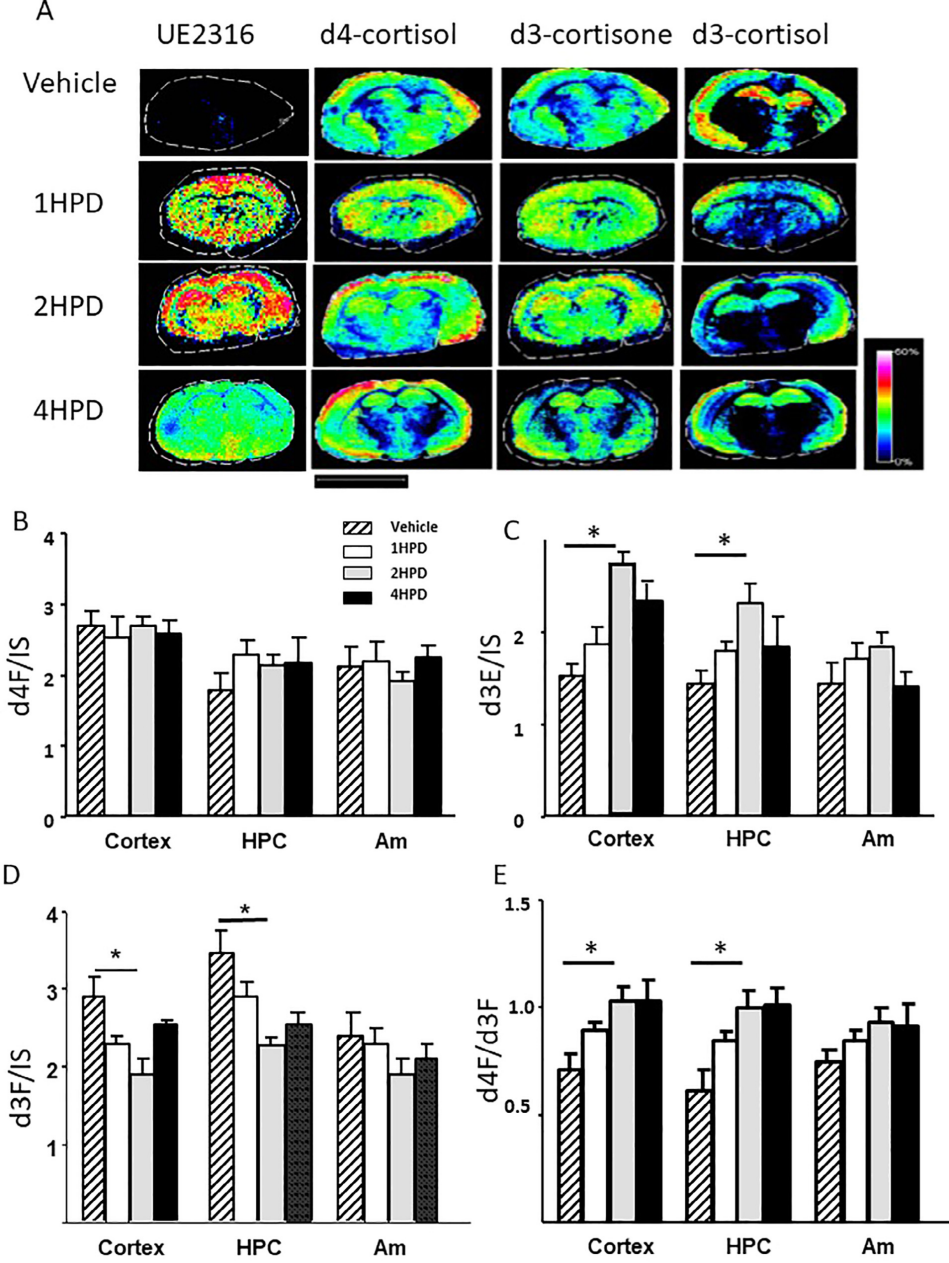
Evaluation of changes in abundances of UE2316, d4-cortisol (d4F), d3-cortisone (d3E) and d3-cortisol (d3F) in brain regions following timed inhibition of 11β-hydroxysteroid dehydrogenase 1 by UE2316, measured by mass spectrometry imaging in sections of murine brain. MS images of (A) UE2316 at *m*/*z* 390.08377, Girard T-d4F at *m*/*z* 480.33073 Da, Girard T-d3E at *m*/*z* 477.29299 Da, Girard T-d3F at *m*/*z* 479.33073 Da, showing that overall signal intensity of the Girard T derivative of d3E increased and that of d3F declined upon treatment with inhibitor. Signal of (C) Girard T-d4F (D) Girard T-d3E (E) Girard T-d3F were normalized to internal standard (IS) d8-corticosterone within regions of interest, cortex, hippocampus (HPC) and amygdala (Am). The ratio of d4F/d3F increased in the cortex and hippocampus over the first 2 h post-dosing (HPD) but not in the amygdala. This effect was sustained to 4 h, although the accumulation of d3E was less marked by 4 h compared with 2 h. Signal intensity is depicted by color on the scale shown. Scale bar (2 mm). Data are mean ± SEM (n = 3) and were compared by a one-way ANOVA with Bonferroni’s post hoc test. ^*^p < 0.05.

By MSI, d4F and its metabolites were observed within hippocampus, cortex and amygdala (Fig. 7). Again UE2316 invoked an increased in d3E and a decline in d3F which reached a maximum at 2 h post-dose and was sustained until 4 h. Corresponding increases in the d4F/d3F ratio were observed which were statistically significant in the hippocampus and cortex.

## 4 Discussion

Although the extensively documented consequences of genetic and pharmacological manipulations of 11β-HSD1 in mice strongly suggest that extra-adrenal regeneration of glucocorticoids is important, the stable-isotope tracer data reported here provide the first *in vivo* quantification of enzyme activity in tissues in rodents. Moreover, although tracer kinetics have been used in combination with arterio-venous sampling to quantify 11β-HSD1 turnover in humans, the data here provide the first information about tracer kinetics within tissues and, notably, within brain sub-regions. Finally, the data presented describe the tissue pharmacokinetics and pharmacodynamic responses of a brain-penetrant 11β-HSD1 inhibitor, confirming the potential for substantial effects on tissue glucocorticoid levels and illustrating the value of MSI in mapping both the drug and metabolite distribution and the consequent changes in tissue steroids.

Previous studies in rodent models have relied on measuring the velocity of 11β-HSD1 in the presence of fixed substrate concentration *ex vivo* in tissue homogenates or slices. While this approach reflects changes in absolute protein concentrations, it cannot be extrapolated to infer the contribution of 11β-HSD1 to active tissues steroids *in vivo*. Moreover, although irreversible inhibitors remain bound in the active site of the enzyme during sample processing, most 11β-HSD1 inhibitors in the development pipeline, including UE2316, are reversible competitive inhibitors that may dissociate in solution. Measurement of ratios of enzyme substrate and product (predominantly 11-dehydrocorticosterone and corticosterone in rodents, cortisone and cortisol in humans) may be more informative, including tissue or microdialysis samples from brain [21], but cannot distinguish glucocorticoids derived from plasma or from local regeneration and reflect only net balance, not turnover, between 11β-HSD1 and 11β-HSD2 activities. Use of stable-isotope tracers *in vivo* can overcome these limitations.

In wild-type mice d4F was infused and readily detected in plasma, along with its metabolites, d3E and d3F. Initial dose-ranging studies were performed to design infusion rates to suppress the hypothalamic-pituitary-adrenal axis, replace adrenal steroids and achieve steady-state circulating d4F concentrations in mouse within the diurnal range of corticosterone. The infusion rate per kg was 300 fold higher than used previously in the clinical setting in man, achieving concentrations ∼40× higher than those studied as human tracers. This suggests a more rapid rate of clearance of glucocorticoids in the mouse than human, by around an order of magnitude.

The calculated production rate of d3F in mice, at ∼120 nmol/h or ∼4800 nmol/kg/h in a 25 g mouse, can then be compared with values in humans of ∼3600 nmol/h or ∼50 nmol/kg/h in a 70 kg man. The substantially higher rate (∼100 fold) of production in mice, will in large part reflect the higher d3E substrate concentrations, around the enzyme *K*_m_. These can only be estimated due to lack of a commercial analytical standard, but can be extrapolated from those of d4F as being around 40-fold higher in mice than in humans. Thus it appears that the turnover of tracer to d3F by murine 11β-HSD1 displays similar kinetics between mouse and human, perhaps ∼2-fold higher in mice, but proceeds in the face of more rapid clearance of tracer by other routes.

In mice with disruption of *Hsd11b1*, d3F was not detected in plasma or in any tissue tested, corroborating the notion that 11β-HSD1 is the only enzyme able to reduce 11-keto-steroids *in vivo* and supporting attenuated formation of the d3F product as an index of pharmacological enzyme inhibition [13]. The levels of d4F achieved in plasma during steady state infusion were the same between wild-type and knockout animals or tended (p = 0.07) to be higher in *Hsd11b1* knockout mice. This may reflect a contribution of dehydrogenase activity of 11β-HSD1 to d4F clearance or indeed changes in other metabolic pathways in the knockout animals that could be teased apart with larger numbers. Lack of reductase activity alone would not influence d4F levels. In humans impaired clearance of glucocorticoids can be detected readily by tracer infusion, seen following 11β-HSD1 inhibition with carbenoxolone [13], a nonselective inhibitor which affects both reductase and dehydrogenase activities. Previous studies of glucocorticoid clearance in mice have generally utilised endogenous glucocorticoids, which are subject to contributions from both oxidative and reductive metabolism. Surprisingly Morgan et al. [29] reported no change in circulating corticosterone concentrations following infusion of the endogenous steroid in mice lacking *Hsd11b1*. This suggests that in mice there may be substantial dehydrogenase as well as reductase activity of 11β-HSD1, which is not present in humans, and these differences in 11β-HSD1 kinetics between mice and humans may explain, at least in part, the inconsistencies between effects of 11β-HSD1 inhibitors in murine models and human patients [4], [30].

Within tissues the highest amounts of tracer detected during d4F infusion were in liver with less detected in brain. Likewise d3E and d3F were easily detected in both tissues, but higher in liver. Ion suppression will vary between tissues, but the intensity of signal of internal standard was not substantially different between liver and brain, suggesting a slower penetration into brain than liver. In each tissue, d4F/d3F ratio was lower than in plasma, consistent with local regeneration of d3F by 11β-HSD1, which was absent in *Hsd11b1* knockout mice. The most striking decrement in tissue versus plasma d4F/d3F ratio was in murine liver, consistent with human data suggesting liver is the major site of cortisol regeneration [31], [32], [33], [34]. Indeed it has been hard to quantify 11β-HSD1 activity in human brain *in vivo* [32].

UE2316 is a brain-penetrant 11β-HSD1 inhibitor that is efficacious in preclinical models [24] and is chemically similar to UE2343, a clinical development candidate [11] to treat Alzheimer’s disease. It selectively inhibits the reductase activity of the enzyme. After a single oral dose, UE2316 was detected rapidly in blood and liver and also in brain, albeit in lower amounts, peaking at 2 h post-dose. This is in keeping with rapid inhibition of enzyme activity measured *ex vivo* in homogenates [24]. During the first 2 h, the drug circulated in plasma at concentrations 10–20-fold in excess of the IC_50_ (measured in stably-transfected cells) [24]. By utilising MSI we have previously demonstrated that UE2316 accesses the brain, distributing widely in many regions [20]. The highest levels were found in hippocampus. Here, we also detected specific metabolites of UE2316 in liver and brain by MSI. Metabolism arose from a mixture of Phase I and II reactions, however different metabolites were abundant in specific subregions of brain, presumably related to the location of specific cytochrome P450 enzymes [35]. Access to this information has important benefits for assessing potential for side-effects and for the duration of the pharmacodynamics effect of the drug within regions of brain important for cognition and memory, such as hippocampus and cortex.

The combination of tracer steroid infusion with MALDI sampling and MSI allowed the presence of UE2316 to be associated with its pharmacodynamic consequences for regeneration of active glucocorticoids. The d4F/d3F ratio in plasma doubled after UE2316 administration, corresponding to halving of whole body regeneration of d3F. Amounts in deuterated tracer and metabolite levels in tissue will in part reflect plasma, but the magnitude of change in dilution of the d4F tracer with tracee (d3F) following administration of drug was greater in both liver and brain than plasma, consistent with local enzyme inhibition. In brain, the extent of inhibition of 11β-HSD1 was in keeping with previous studies, measured *ex vivo* in brain homogenates [24]. However, suppression of glucocorticoid regeneration was sustained for longer in brain than liver, despite a similar profile in amounts of drug with time. This might reflect slower turnover of steroid within brain tissue. Within the brain subtle differences in the extent of inhibition between regions could also be seen. Inhibition of 11β-HSD1 by UE2316 was not detected in amygdala but was achieved in hippocampus and cortex, regions with higher enzyme activity. Again this demonstrates that regional-selective responses to drugs might be assessed by the approach described here. The hippocampal region is vitally important in age-related memory loss and its susceptibility to corticosteroid action is well established. Inhibition of 11β-HSD1 in brain has previously been shown to be effective in slowing disease progression in murine models of Alzheimer’s disease [24].

In summary, combined pharmacokinetic and pharmacodynamics analysis using tracer infusion and MSI has shown that the turnover of 11β-HSD1 reductase activity in mice is rapid *in vivo*, and occurs in liver and brain, whereas it is absent in *Hsd11b1*-deficient mice and is measurably inhibited by UE2316. These insights inform the extrapolation of results from mice to humans and justify continued efforts to target 11β-HSD1 in the brain.
